# A window to the amygdala: concurrent encoding of choice preference in multi-unit activity in the amygdala and in eye movements

**DOI:** 10.1186/1471-2202-12-S1-O13

**Published:** 2011-07-18

**Authors:** Christopher K Kovach, Rick L Jenison

**Affiliations:** 1 Department of Neurosurgery, University of Iowa, Iowa City, IA, 52245, USA; 2 Department of Psychology, University of Wisconsin, Madison, WI, 53706, USA

## 

**Figure 1:**
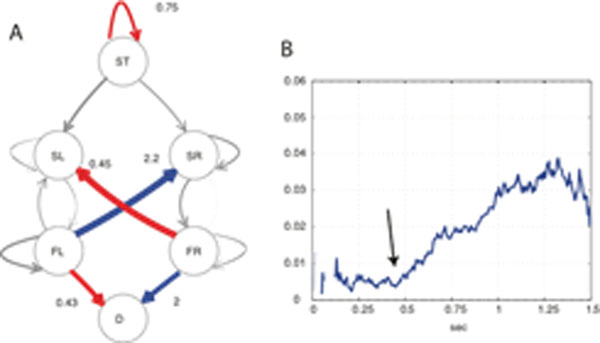
A Markov model of eye movement during a two-alternative choice task. **A:** Modeled states are start of trial (ST), saccade-to-left item (SL), saccade-to-right item (SR), fixate-on-left item (FL), fixate-on-right item (FR), decision (D). Multinomial logistic regression reveals an interaction between transition probabilities and choice side (P < .001). Significantly increasing (blue) and decreasing (red) changes in transition probability show an increase in saccades towards the chosen item and in choosing the item fixated last in the trial. **B.** The time course of average mutal information reveals to the onset of information accumulation at 500 ms after stimulus onset.

Research on the functions of the amygdala has pointed towards multifaceted roles in learning, emotion, attention and decision making. The degree to which the various functions of the amygdala reflect different aspects of a common set of operations, such as the modulation of attention, remains unknown. A recent observation links deficits in facial expressions following bilateral amygdala damage to a specific attentional mechanism, reflected in an abnormal pattern of gaze directed to faces [1], raising the question whether other aspects of amygdala function can similarly be traced to the modulation of attention. For example, when choosing a preferred item from multiple alternatives, eye movements reveal the emergence of preference for one of the [3,4], suggesting an integral role for attention in the formation of choice preference, whose nature, however, remains uncertain. We consider whether preference-related modulation of eye movement depends on encoding of value in amygdala responses, which has recently been described during economic decision-making in humans [2]. Using concurrent eye tracking and recordings from the amygdala we aim to compare the time-course of information about choice encoded in unit activity with that encoded in patterns of gaze. To provide a framework for this comparison we combine a novel and sensitive generalized linear modeling approach to eye movement analysis with Bayesian particle filtering, and apply it towards identifying the time-evolution of information contained in eye movements, which will be compared with spike-encoding using equivalent procedures on spiking activity. For each of 4 blocks of 138 trials, a Markov model of eye movement transitions (Fig 1A) was fitted to a subset of data which excluded that trial. Choice outcome was then predicted at each time point in the trial using a sequential Bayesian particle filter from likelihoods generated by the fitted model. The filter was considered to favor the right option over the left when the proportion of particles favoring right exceeded some threshold. The quality of the prediction was quantified at each time point using mutual information between predicted and observed outcomes across multiple thresholds. Averaging over thresholds reduces noise variance in the estimate of MI, allowing for a clearer representation of the time course of the prediction quality (Fig 1B). This procedure reveals that Information about choice in eye-movements appears with a monotonically increasing trend having an abrupt onset around 500 ms after the stimulus onset. The emergence of information in eye movements therefore resembles the previously reported time course of value encoding in amygdala neurons [2], implying that value encoding in the amygdala and preference-related biases of gaze emerge concurrently. Further work will focus on the direct comparison of information in simultaneously recorded sets of eyetracking data and multiunit activity.
